# Acute ischemic stroke: a rare complication of hump-nosed pit viper (*Hypnale* spp.) bite: a case report

**DOI:** 10.1186/s13256-022-03442-3

**Published:** 2022-06-04

**Authors:** R. M. M. K. Namal Rathnayaka, P. E. A. Nishanthi Ranathunga, S. A. M. Kularatne, Sanath Jayasinghe

**Affiliations:** 1 Department of Pharmacology, Faculty of Medicine, Sabaragamuwa University of Sri Lanka, Hidellana, Ratnapura, Sri Lanka; 2 Department of Veterinary Pathobiology, Faculty of Veterinary Medicine and Animal Science, University of Peradeniya, Peradeniya, Sri Lanka; 3 Intensive Care Unit, Teaching Hospital, Ratnapura, Sri Lanka; 4 Medical Unit, Teaching Hospital, Ratnapura, Sri Lanka; 5 Department of Medicine, Faculty of Medicine, University of Peradeniya, Peradeniya, Sri Lanka; 6 No. 11, Flower Road, New Town Housing Scheme 01, New Town, Ratnapura, Sri Lanka

**Keywords:** Ischemic stroke, Hump-nosed pit viper, *Hypnale*, Snakebites, Sri Lanka

## Abstract

**Background:**

Hump-nosed pit viper is a medically important deadly venomous snake in Sri Lanka and is the commonest cause of venomous snakebites in the country. It frequently causes local effects and systemic manifestations such as acute kidney injury and coagulopathy that occur in less than 10% of all bites. This also includes some atypical presentations such as thrombotic microangiopathy and myocardial infarction. Currently, no antivenom is available for hump-nosed pit viper bites in Sri Lanka, and patients are managed with supportive treatment. This case illustrates an acute ischemic stroke following a hump-nosed viper bite, which is the second case in the literature.

**Case presentation:**

A 71-year-old a Sinhalese male patient presented with left-sided hemiparesis with mouth deviation on day 2 of hump-nosed viper (*Hypnale* spp.) bite on the right foot. Non-contrast computed tomography of brain showed right ischemic stroke in internal capsule. He was given antiplatelets and statins and continued supportive treatment including limb physiotherapy and speech therapy. He recovered completely and was discharged on day 4 with clinic follow-up.

**Conclusions:**

Physicians should be aware that ischemic cerebral infarcts may occur following hump-nosed pit viper bites.

## Background

**Fig. 1:**
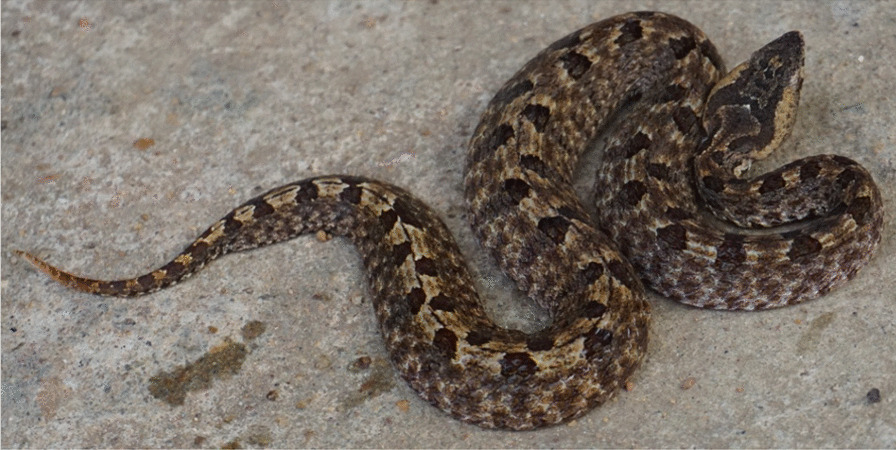
Hump-nosed pit viper (*Hypnale hypnale*)

Hump-nosed pit viper is a medically important deadly venomous snake in Sri Lanka (Fig. 1) and is the commonest cause of venomous snakebites in the country (22–77%) [1]. It frequently causes local envenoming and less commonly cause systemic effects such as acute kidney injury and venom-induced consumption coagulopathy (VICC) [2, 3]. Myocardial infarction (MI) [4], Kounis syndrome [5], microangiopathic hemolysis [6], thrombotic microangiopathy (TMA), hemolytic uremic syndrome (HUS), and thrombotic thrombocytopenic purpura (TTP) [2, 7] are some atypical presentations of hump-nosed pit viper bites. Currently, no antivenom is available for *Hypnale* bites in Sri Lanka or India, and supportive treatments are carried out for these bites. We describe such a presentation of acute ischemic stroke following a bite by hump-nosed viper as the second reported case of Sri Lanka.

## Case presentation

A 71-year old previously well Sinhalese male was transferred from a local hospital for further management of weakness of left upper and lower limbs following a hump-nosed pit viper bite. At a local hospital, his 20 min whole blood clotting test (WBCT20) was normal. On the previous day, while the patient was working in the home garden at about 11:00, his right foot was bitten by a snake identified by the patient and the relatives as a hump-nosed pit viper. However, the snake was not caught or killed for religious reasons. When he was shown a formalin-preserved specimen of hump-nosed viper at the ward, he identified and confirmed the offending snake. He was given native treatment (local application and decoction) once and was admitted to a local hospital. The next day, he developed weakness of left lower and upper limbs at about 11:30. His urine output was normal, and there was moderate pain at site of bite. On admission to the tertiary care center, his Glasgow coma scale (GCS) was 15/15, his mouth deviated to the right, and he had reduced muscle power in both left upper and lower limbs (3/5) with exaggerated tendon reflexes. Ptosis or external ophthalmoplegia was not observed. Also, there was slurring of speech. However, there was no sensory impairment.

**Fig. 2:**
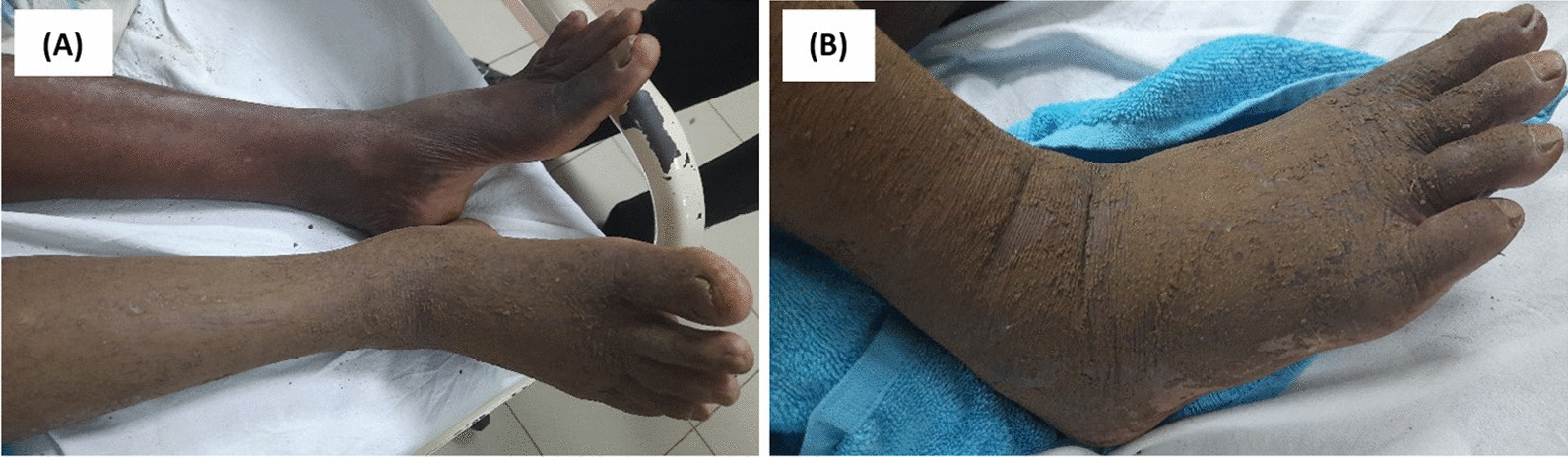
**A** Site of bite—right foot at day 2 of snakebite. (**B**) Local application of native herbal medicine

**Fig. 3:**
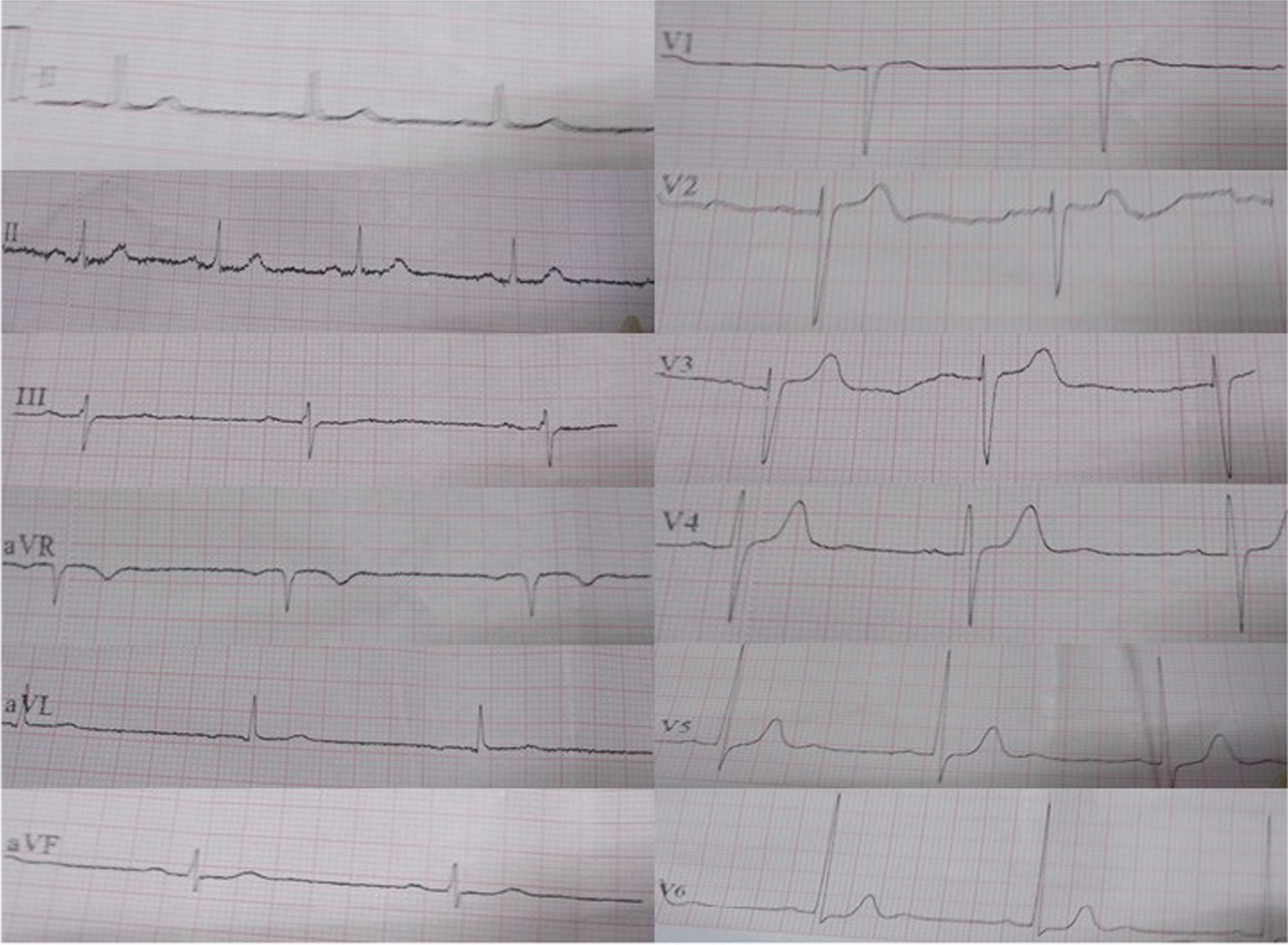
Electrocardiogram showing bradycardia of the patient on day 2 of snakebite

**Fig. 4:**
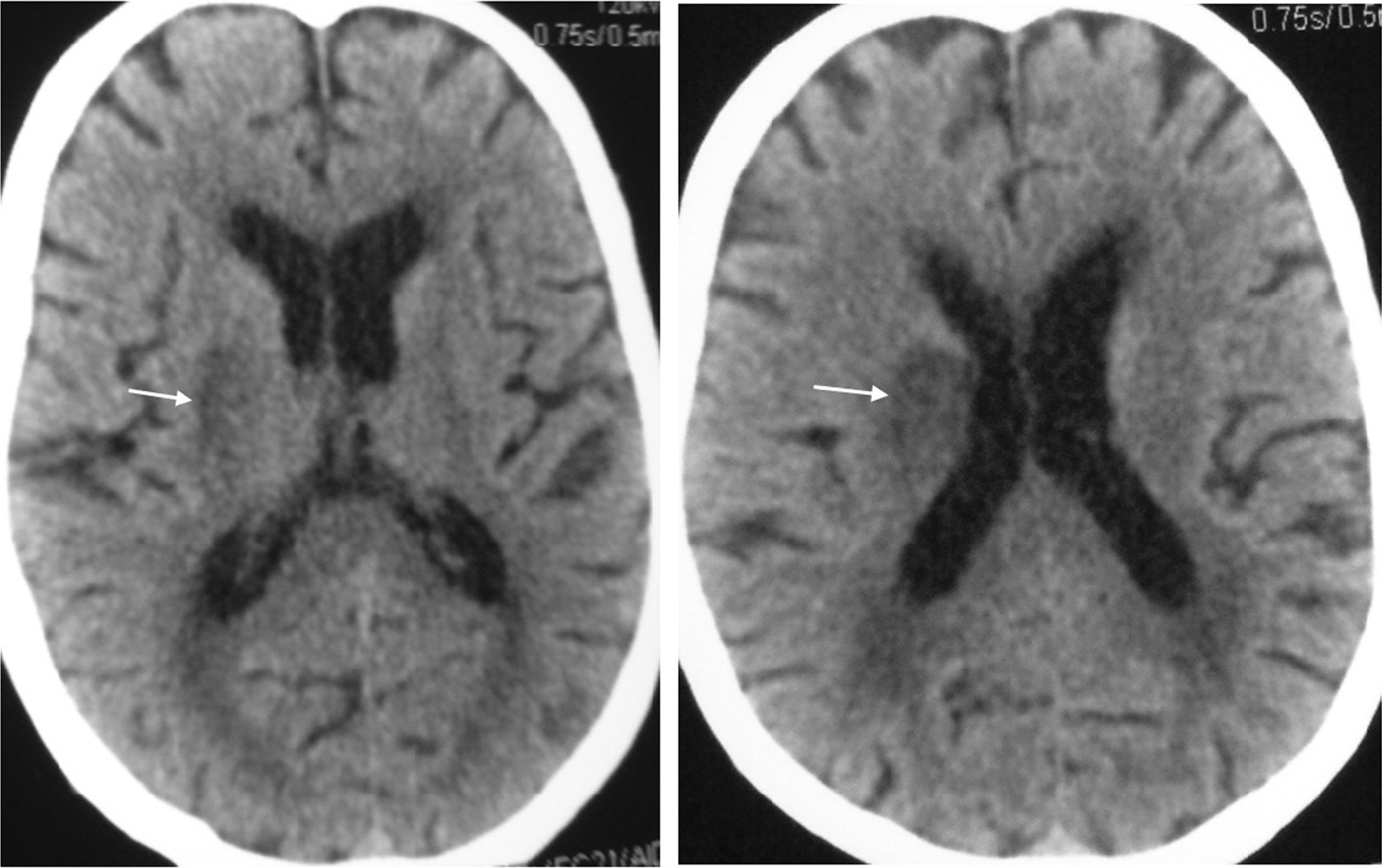
Computed tomography scan of brain showing an ischemic infarct in right-sided internal capsule (arrow)

There was moderate swelling over the bitten foot, and no necrosis was observed. Fang punctures were difficult to identify owing to local application of herbal medicine over the bitten limb (Fig. 2). His blood pressure was 160/80 mmHg, pulse rate was 52 beats per minute, and O_2_ saturation was 97% on room air. Twenty-minute whole blood clotting test done three times 6 hours apart was normal, and the other laboratory findings are presented in Table 1. Electrocardiogram (ECG) showed bradycardia (Fig. 3). Blood picture and chest X-ray were normal. The non-contrast computed tomography scan of brain on admission (day 2 of snakebite) was normal, but on day 3 it showed an ischemic infarct in right internal capsule (Fig. 4).

**Table 1:** Laboratory findings of the patient

Investigation	Reference range	Day 1	Day 2	Day 3
WBC (×10^3^ µL^−1^)	4–10	10	10	9.8
Neutrophils (%)	50–70	31	34	35
Neutrophil count (µL^−1^)	2000–7000	3210	3430	3440
Lymphocytes (%)	20–40	64	61	59
Lymphocyte count (µL^−1^)	800–4000	6590	6210	5830
Platelets (×10^3^ µL^−1^)	150–400	195	167	175
Hb (g dL^−1^)	11–16	10.4	9.7	9.9
PT (seconds)	10–15	11.4/12	12.2/12	
INR	1–1.4	0.95	1.02	
aPTT (seconds)	25–30	29/30		
Na^+^ (mmol L^−1^)	135–145	144	141	137
K^+^ (mmol L^−1^)	3.5–4.5	4	4	3.8
Blood urea (mmol L^−1^)	2.2–8.2	2.5	3.5	7
Creatinine (μmol L^−1^)	60–115	128	113	89
SGOT (AST) (U I^−1^)	0–35	33		30
SGPT (ALT) (U I^−1^)	0–45	41		22
CRP (mg L^−1^)	< 6	< 5	< 5	8

*WBC* white blood cells, *Hb* hemoglobin, *PT* prothrombin time, *INR* international normalized ratio, *aPTT* activated partial thromboplastin time, *Na*^+^ sodium, *K*^+^ potassium, *SGOT* serum glutamic oxaloacetic transaminase, *AST* aspartate aminotransferase, *SGPT* serum glutamic pyruvic transaminase, *ALT* alanine aminotransferase, *CRP* C reactive protein

The patient was started on aspirin 300 mg, clopidogrel 75 mg, and atorvastatin 20 mg *nocte* after giving loading doses of those medications. Intravenous clindamycin 600 mg 6-hourly and cefotaxime 1 g 8-hourly were administered. He continued limb physiotherapy and speech therapy. He was discharged on day 4 of snakebite by arranging clinic visits with a plan for stroke rehabilitation (physiotherapy) with aspirin and atorvastatin. At first clinic visit 2 weeks after the discharge, his limb weakness and pulse rate were improved. His lipid profile was normal.

## Discussion

Ischemic stroke following hump-nosed pit viper bite has previously been reported in a Sri Lankan patient who developed left-sided hemiparesis associated with acute kidney injury [8]. *In vitro* studies confirmed that *Hypnale* venom has potent cytotoxic, mild procoagulant, weak neurotoxic, and myotoxic activity [9]. However, procoagulant toxins of *Hypnale* venom have thus far not been isolated and characterized, and it is hypothesized that the procoagulant activity is due to thrombin-like enzymes (TLEs) of the venom [10], which may have caused the cerebral infarction in our patient. Thrombin-like enzymes activate clotting pathways, leading to the formation of thrombi, and hence deposit them as fibrin, causing vascular occlusion, which results in reduction of blood supply to the organ. This commonly occurs in vessels of kidneys, brain, heart, or pituitary gland. When red blood cells flow through these blocked vessels, they get distorted, which can be seen on peripheral blood microscopy as schistocytes (fragmented red blood cells). This condition is called microangiopathic hemolysis and can be observed in *Hypnale* bites [6]. When this organ ischemia occurs together with thrombocytopenia and microangiopathic hemolysis, the process is called TMA, which can also be observed in hump-nosed pit viper bites [7]. It was evidenced that, in our patient, cerebral infarction was due to the venom because he also had other venom effects, including bradycardia, mild reduction of hemoglobin (Hb), and mild elevation of creatinine. While thrombi are formed, circulating platelets can get trapped in fibrin mesh, resulting in thrombocytopenia as our patient’s platelet count on day 2 was near to the lower marginal limit (Table 1). As a drug therapy, these patients can be administered aspirin after the clotting abnormality is resolved, and physiotherapy plays a vital part in the management. To determine whether the type of snake venom is hemotoxic, neurotoxic, nephrotoxic, or myotoxic, there are various investigations that can be done according to the severity of the patient’s condition. Coagulopathy is detected from the clotting profile, including prothrombin time (PT)/international normalized ratio (INR), activated partial thromboplastin time (aPTT), WBCT20, fibrinogen, and D-dimers. Nephrotoxicity is detected from serum creatinine, blood urea, and renal ultrasound scan. Myotoxicity is assessed using laboratory findings related to rhabdomyolysis such as myoglobinuria, serum K^+^, and creatine phosphokinase.

Interestingly, the clotting profile of our patient was normal throughout (Table 1), and it could be hypothesized that he has depletion of the downstream clotting factors due to consumption and maintained factor levels marginally sufficient for normal clotting. At this stage, fibrinogen levels could be reduced. However, we were unable to measure fibrinogen levels owing to lack of that facility at the hospital. In Sri Lanka, both hemorrhagic and ischemic cerebral infarction is common with Russell’s viper (*Daboia russelii*) envenoming, and sometimes these may be fatal [11]. Cardiac effects such as MI, atrial fibrillation, acute ischemic changes, bradycardia, and cardiac arrest have previously been reported following hump-nosed pit viper bites [4, 12]. Our patient had bradycardia throughout. However, we expect heart rate to be high because of the local pain. Therefore, reduction of heart rate may be the effect of venom on heart, as previously suggested [12]. Initially, it was thought that bradycardia might also be due to the patient’s old age. However, his pulse rate improved to around 70 beats per minute 4 days after the snakebite. Our patient had pure motor involvement of arms, face, and legs, which was suggestive of ischemia mainly to the posterior limb of internal capsule and involvement of middle cerebral artery. Even though strokes are very rare in *Hypnale* bites, physicians should be aware of them, because as there is no antivenom, these patients should be managed with available supportive care. Otherwise, they may be administered available antivenom unnecessarily.

## Conclusions

Physicians should be aware that ischemic cerebral infarcts may occur following hump-nosed pit viper bites.

## Data Availability

All the data generated and analyzed for this case report have been included in this article.

## Abbreviations

MI: Myocardial infarction
VICC: Venom-induced consumption coagulopathy
HUS: Hemolytic uremic syndrome
TTP: Thrombotic thrombocytopenic purpura
TMA: Thrombotic microangiopathy
GCS: Glasgow coma scale
ECG: Electrocardiogram
TLEs: Thrombin-like enzymes
Hb: Hemoglobin
